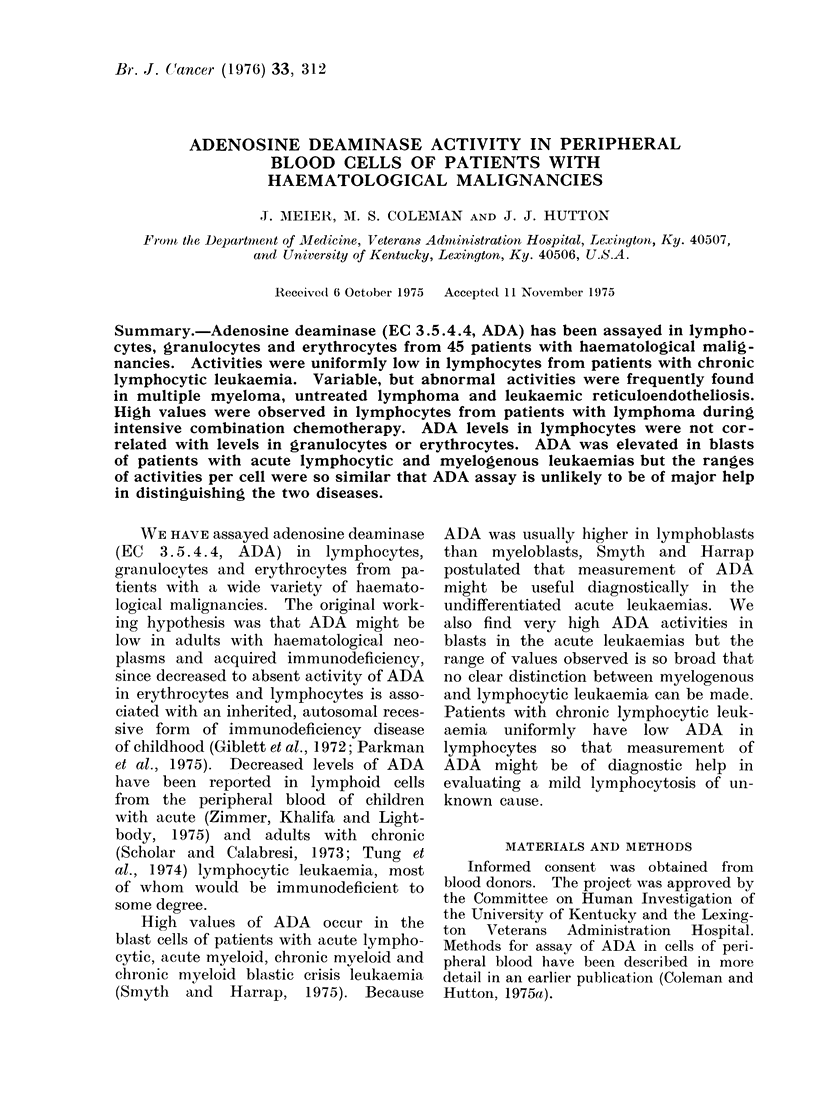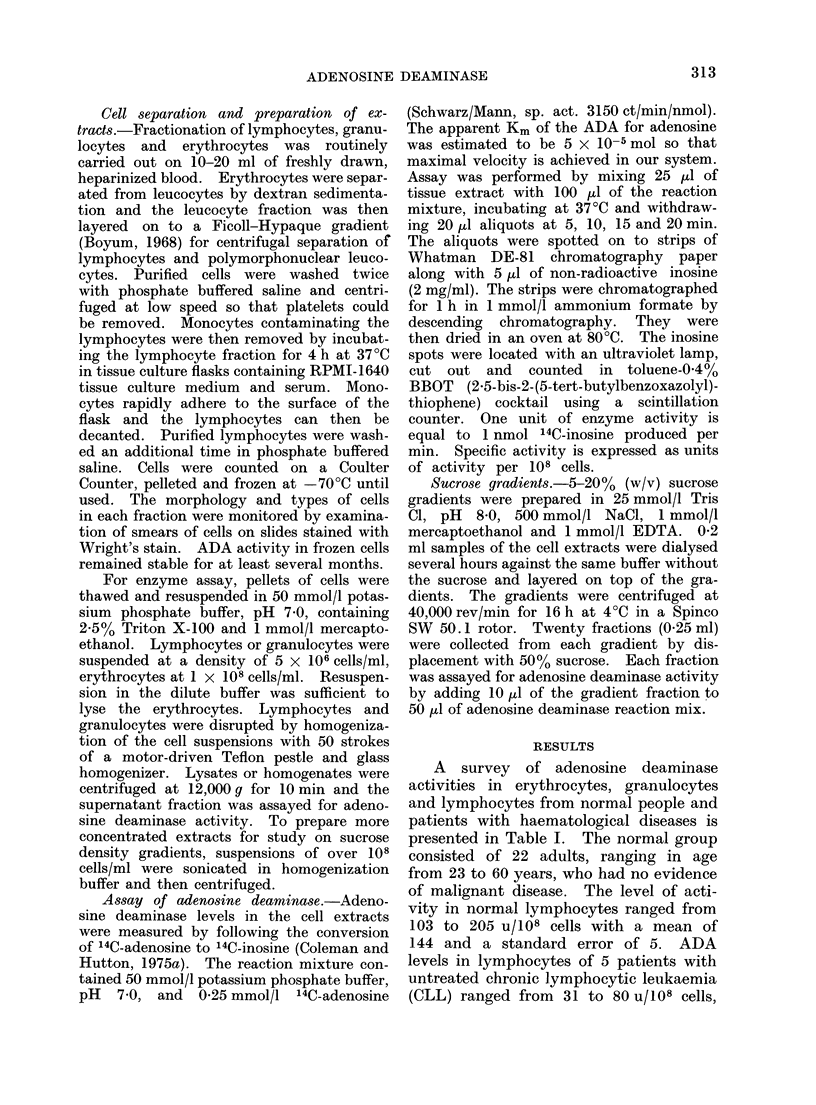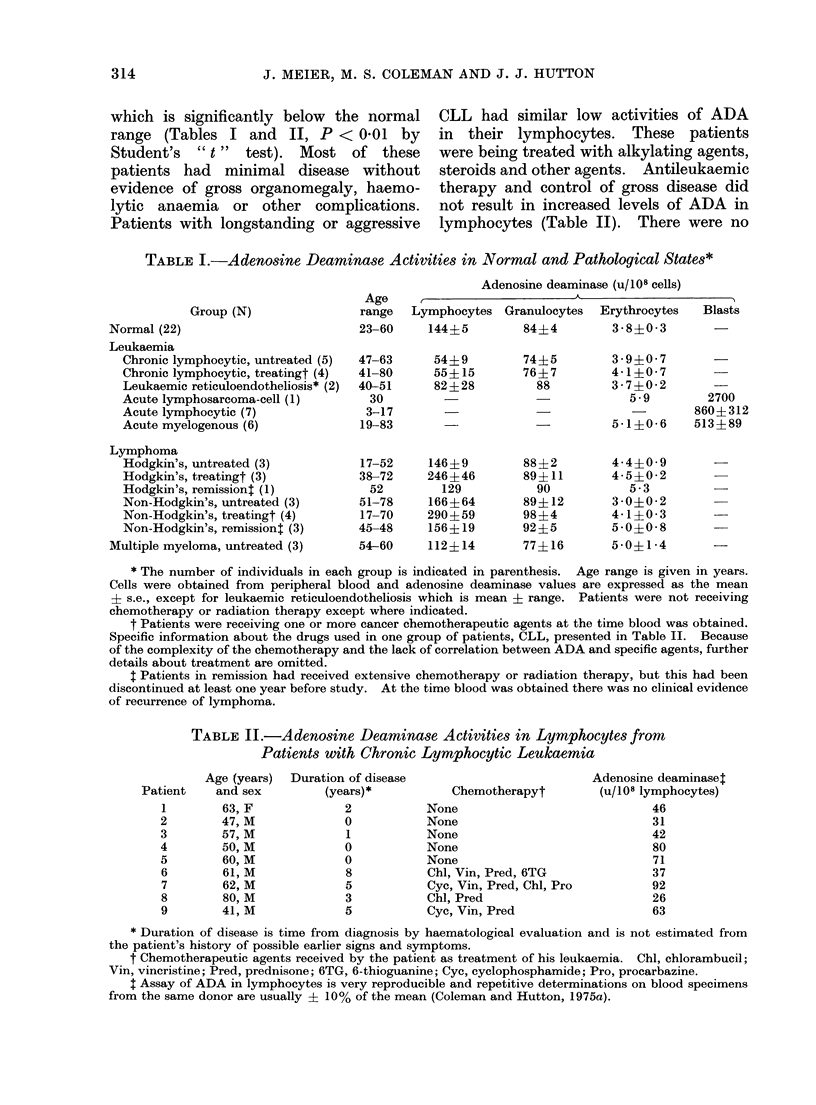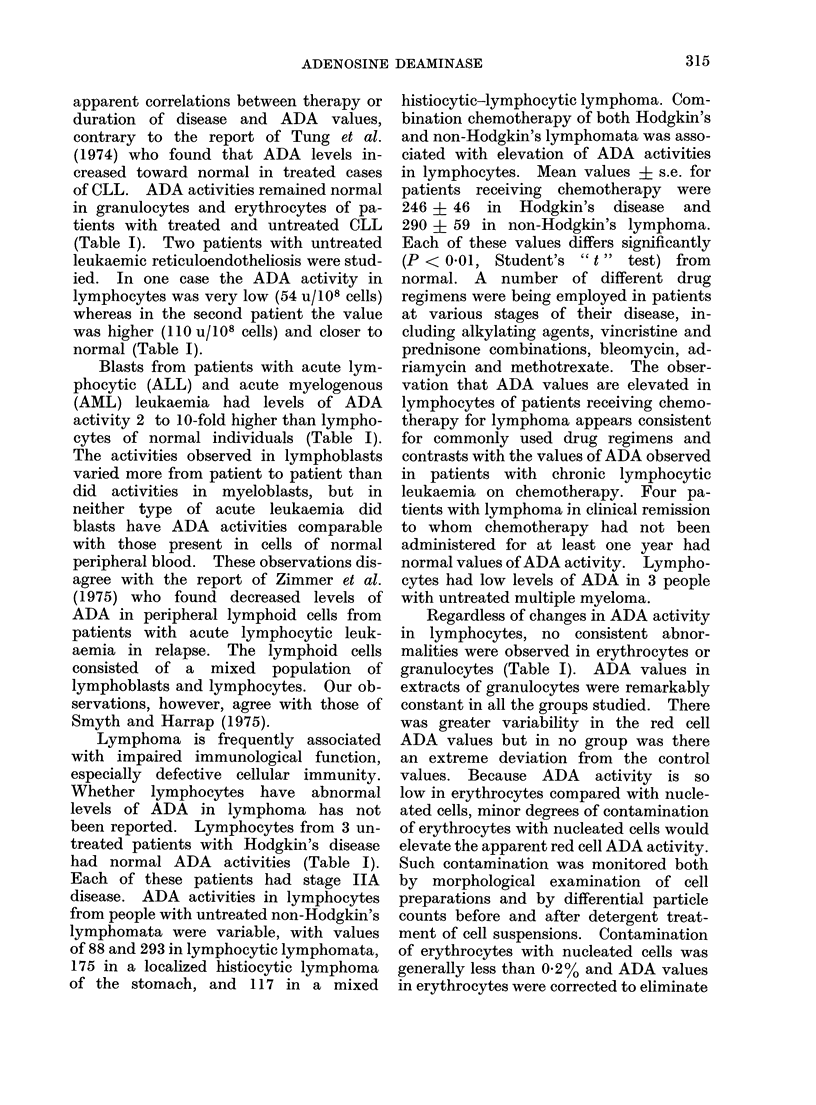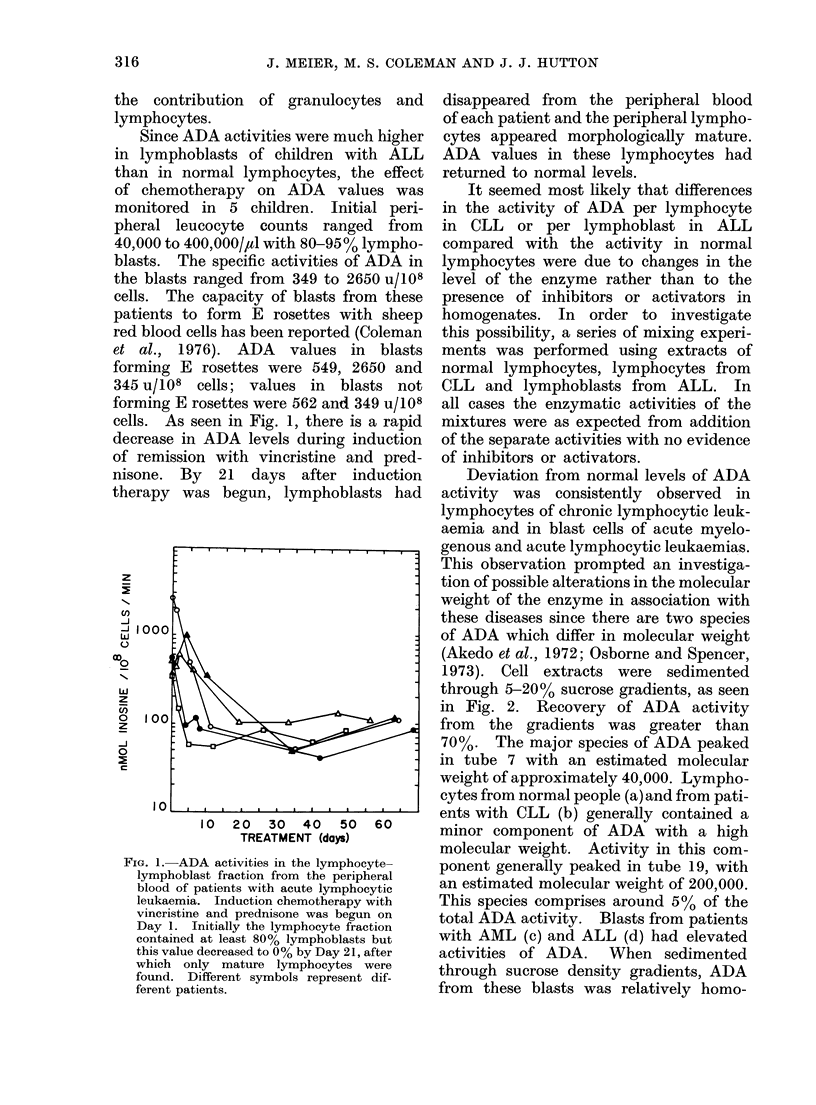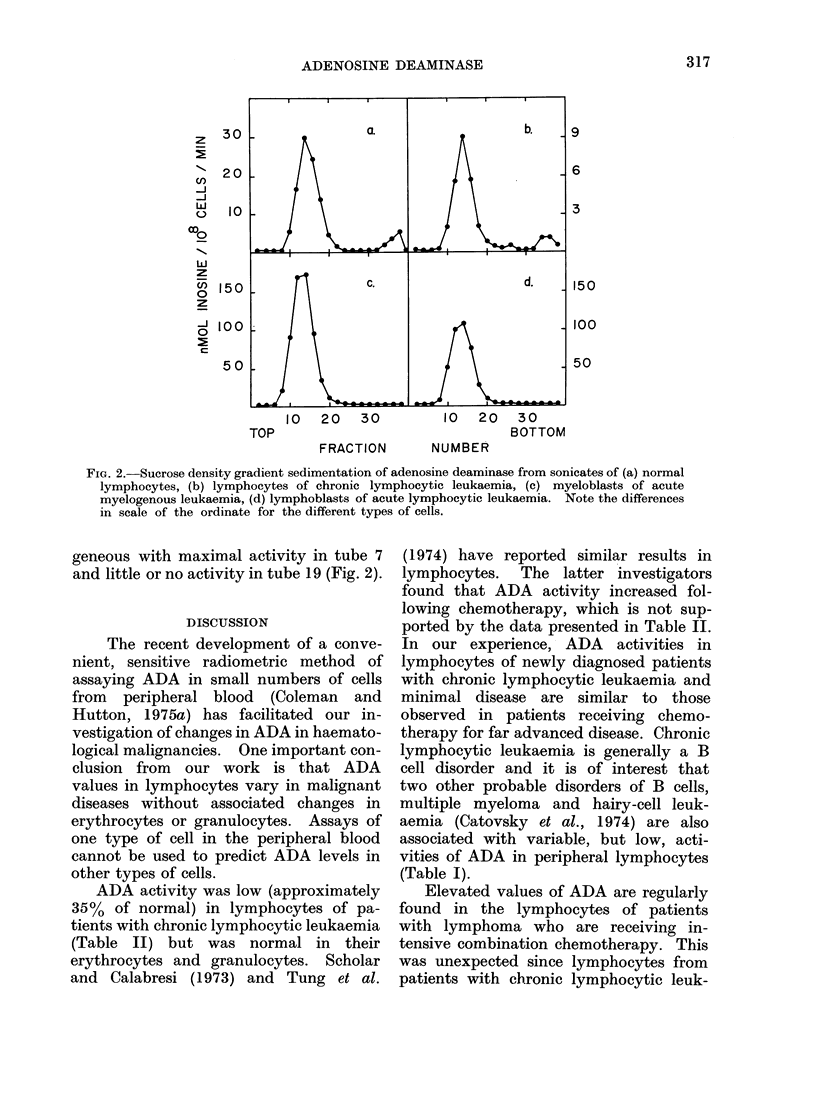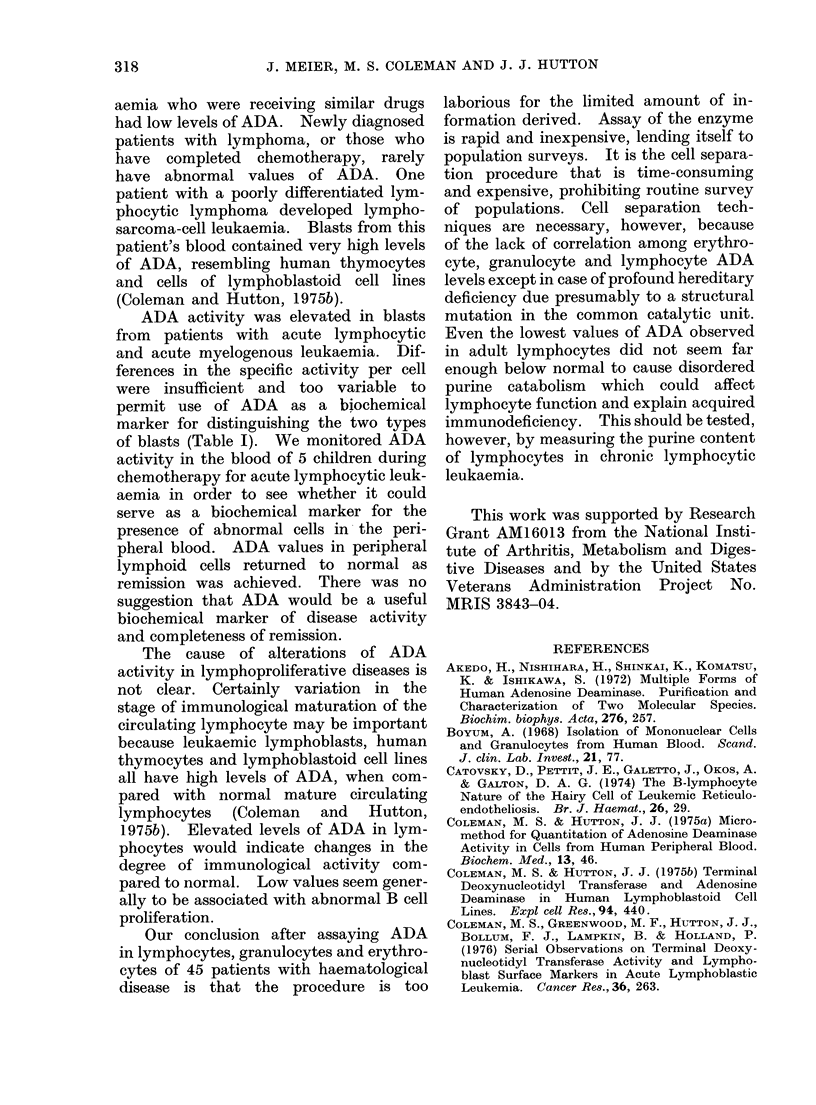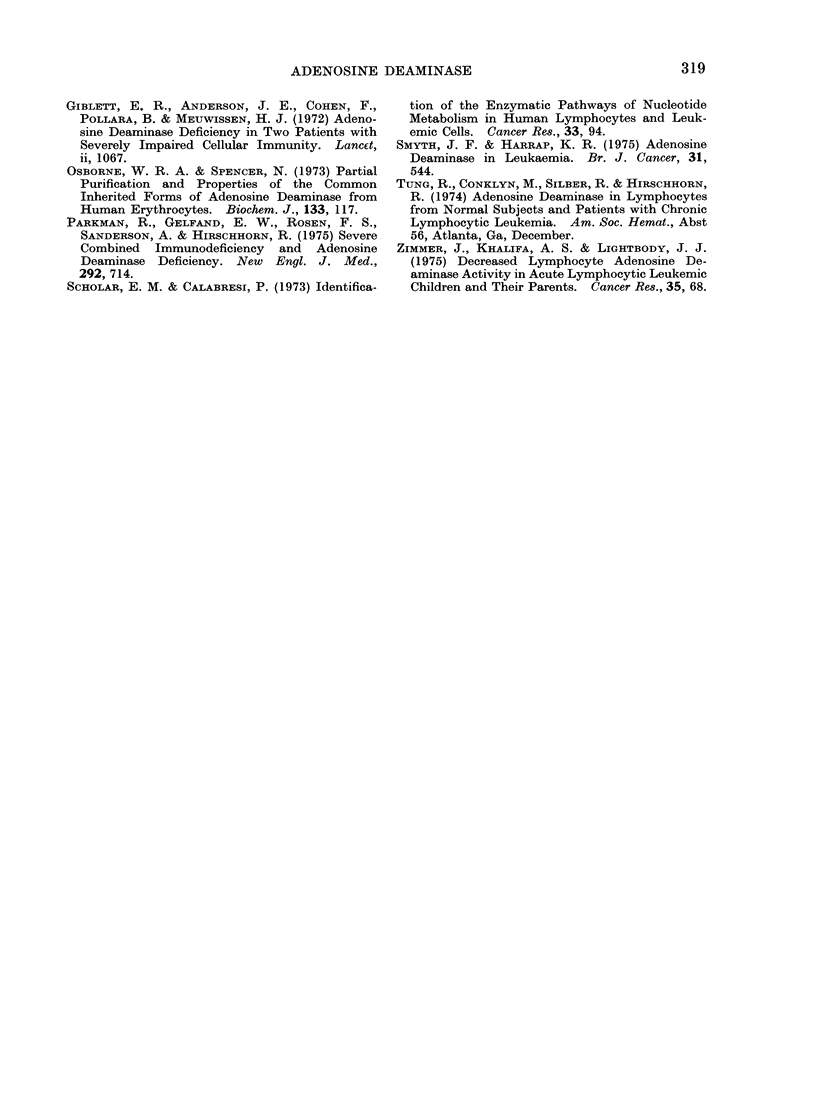# Adenosine deaminase activity in peripheral blood cells of patients with haematological malignancies.

**DOI:** 10.1038/bjc.1976.45

**Published:** 1976-03

**Authors:** J. Meier, M. S. Coleman, J. J. Hutton

## Abstract

Adenosine deaminase (EC 3.5.4.4, ADA) has been assayed in lymphocytes, granulocytes and erythrocytes from 45 patients with haematological malignancies. Activities were uniformly low in lymphocytes from patients with chronic lymphocytic leukaemia. Variable, but abnormal activities were frequently found in multiple myeloma, untreated lymphoma and leukaemic reticuloendotheliosis. High values were observed in lymphocytes from patients with lymphoma during intensive combination chemotherapy. ADA levels in lymphocytes were not correlated with levels in granulocytes or erythrocytes. ADA was elevated in blasts of patients with acute lymphocytic and myelogenous leukaemias but the ranges of activities per cell were so similar that ADA assay is unlikely to be of major help in distinguishing the two diseases.


*Br. J. Cancer* (1976) **33**, 312

### ADENOSINE DEAMINASE ACTIVITY IN PERIPHERAL BLOOD CELLS OF PATIENTS WITH HAEMATOLOGICAL MALIGNANCIES

J. MEIER, M. S. COLEMAN AND J. J. HUTTON

*From the Department of Medicine, Veterans Administration Hospital, Lexington, Ky.* 40507, *and University of Kentucky, Lexington, Ky.* 40506, *U.S.A.*

Received 6 October 1975 Accepted 11 November 1975

**Summary.—Adenosine deaminase (EC 3.5.4.4, ADA) has been assayed in lymphocytes, granulocytes and erythrocytes from 45 patients with haematological malignancies. Activities were uniformly low in lymphocytes from patients with chronic lymphocytic leukaemia. Variable, but abnormal activities were frequently found in multiple myeloma, untreated lymphoma and leukaemic reticuloendotheliosis. High values were observed in lymphocytes from patients with lymphoma during intensive combination chemotherapy. ADA levels in lymphocytes were not correlated with levels in granulocytes or erythrocytes. ADA was elevated in blasts of patients with acute lymphocytic and myelogenous leukaemias but the ranges of activities per cell were so similar that ADA assay is unlikely to be of major help in distinguishing the two diseases.**

WE HAVE assayed adenosine deaminase (EC 3.5.4.4, ADA) in lymphocytes, granulocytes and erythrocytes from patients with a wide variety of haematological malignancies. The original working hypothesis was that ADA might be low in adults with haematological neoplasms and acquired immunodeficiency, since decreased to absent activity of ADA in erythrocytes and lymphocytes is associated with an inherited, autosomal recessive form of immunodeficiency disease of childhood (Giblett *et al.*, 1972; Parkman *et al.*, 1975). Decreased levels of ADA have been reported in lymphoid cells from the peripheral blood of children with acute (Zimmer, Khalifa and Lightbody, 1975) and adults with chronic (Scholar and Calabresi, 1973; Tung *et al.*, 1974) lymphocytic leukaemia, most of whom would be immunodeficient to some degree.

High values of ADA occur in the blast cells of patients with acute lymphocytic, acute myeloid, chronic myeloid and chronic myeloid blastic crisis leukaemia (Smyth and Harrap, 1975). Because ADA was usually higher in lymphoblasts than myeloblasts, Smyth and Harrap postulated that measurement of ADA might be useful diagnostically in the undifferentiated acute leukaemias. We also find very high ADA activities in blasts in the acute leukaemias but the range of values observed is so broad that no clear distinction between myelogenous and lymphocytic leukaemia can be made. Patients with chronic lymphocytic leukaemia uniformly have low ADA in lymphocytes so that measurement of ADA might be of diagnostic help in evaluating a mild lymphocytosis of unknown cause.

### MATERIALS AND METHODS

Informed consent was obtained from blood donors. The project was approved by the Committee on Human Investigation of the University of Kentucky and the Lexington Veterans Administration Hospital. Methods for assay of ADA in cells of peripheral blood have been described in more detail in an earlier publication (Coleman and Hutton, 1975*a*).

*Cell separation and preparation of extracts.*—Fractionation of lymphocytes, granulocytes and erythrocytes was routinely carried out on 10–20 ml of freshly drawn, heparinized blood. Erythrocytes were separated from leucocytes by dextran sedimentation and the leucocyte fraction was then layered on to a Ficoll–Hypaque gradient (Boyum, 1968) for centrifugal separation of lymphocytes and polymorphonuclear leucocytes. Purified cells were washed twice with phosphate buffered saline and centrifuged at low speed so that platelets could be removed. Monocytes contaminating the lymphocytes were then removed by incubating the lymphocyte fraction for 4 h at 37°C in tissue culture flasks containing RPMI-1640 tissue culture medium and serum. Monocytes rapidly adhere to the surface of the flask and the lymphocytes can then be decanted. Purified lymphocytes were washed an additional time in phosphate buffered saline. Cells were counted on a Coulter Counter, pelleted and frozen at −70°C until used. The morphology and types of cells in each fraction were monitored by examination of smears of cells on slides stained with Wright's stain. ADA activity in frozen cells remained stable for at least several months.

For enzyme assay, pellets of cells were thawed and resuspended in 50 mmol/l potassium phosphate buffer, pH 7·0, containing 2·5% Triton X-100 and 1 mmol/l mercaptoethanol. Lymphocytes or granulocytes were suspended at a density of $5 \times 10^6$ cells/ml, erythrocytes at $1 \times 10^8$ cells/ml. Resuspension in the dilute buffer was sufficient to lyse the erythrocytes. Lymphocytes and granulocytes were disrupted by homogenization of the cell suspensions with 50 strokes of a motor-driven Teflon pestle and glass homogenizer. Lysates or homogenates were centrifuged at 12,000 *g* for 10 min and the supernatant fraction was assayed for adenosine deaminase activity. To prepare more concentrated extracts for study on sucrose density gradients, suspensions of over $10^8$ cells/ml were sonicated in homogenization buffer and then centrifuged.

*Assay of adenosine deaminase.*—Adenosine deaminase levels in the cell extracts were measured by following the conversion of $^{14}$C-adenosine to $^{14}$C-inosine (Coleman and Hutton, 1975*a*). The reaction mixture contained 50 mmol/l potassium phosphate buffer, pH 7·0, and 0·25 mmol/l $^{14}$C-adenosine (Schwarz/Mann, sp. act. 3150 ct/min/nmol). The apparent $K_m$ of the ADA for adenosine was estimated to be $5 \times 10^{-5}$ mol so that maximal velocity is achieved in our system. Assay was performed by mixing 25 μl of tissue extract with 100 μl of the reaction mixture, incubating at 37°C and withdrawing 20 μl aliquots at 5, 10, 15 and 20 min. The aliquots were spotted on to strips of Whatman DE-81 chromatography paper along with 5 μl of non-radioactive inosine (2 mg/ml). The strips were chromatographed for 1 h in 1 mmol/l ammonium formate by descending chromatography. They were then dried in an oven at 80°C. The inosine spots were located with an ultraviolet lamp, cut out and counted in toluene-0·4% BBOT (2·5-bis-2-(5-tert-butylbenzoxazolyl)-thiophene) cocktail using a scintillation counter. One unit of enzyme activity is equal to 1 nmol $^{14}$C-inosine produced per min. Specific activity is expressed as units of activity per $10^8$ cells.

*Sucrose gradients.*—5–20% (w/v) sucrose gradients were prepared in 25 mmol/l Tris Cl, pH 8·0, 500 mmol/l NaCl, 1 mmol/l mercaptoethanol and 1 mmol/l EDTA. 0·2 ml samples of the cell extracts were dialysed several hours against the same buffer without the sucrose and layered on top of the gradients. The gradients were centrifuged at 40,000 rev/min for 16 h at 4°C in a Spinco SW 50.1 rotor. Twenty fractions (0·25 ml) were collected from each gradient by displacement with 50% sucrose. Each fraction was assayed for adenosine deaminase activity by adding 10 μl of the gradient fraction to 50 μl of adenosine deaminase reaction mix.

### RESULTS

A survey of adenosine deaminase activities in erythrocytes, granulocytes and lymphocytes from normal people and patients with haematological diseases is presented in Table I. The normal group consisted of 22 adults, ranging in age from 23 to 60 years, who had no evidence of malignant disease. The level of activity in normal lymphocytes ranged from 103 to 205 u/$10^8$ cells with a mean of 144 and a standard error of 5. ADA levels in lymphocytes of 5 patients with untreated chronic lymphocytic leukaemia (CLL) ranged from 31 to 80 u/$10^8$ cells, which is significantly below the normal range (Tables I and II, $P < 0{\cdot}01$ by Student's "$t$" test). Most of these patients had minimal disease without evidence of gross organomegaly, haemolytic anaemia or other complications. Patients with longstanding or aggressive CLL had similar low activities of ADA in their lymphocytes. These patients were being treated with alkylating agents, steroids and other agents. Antileukaemic therapy and control of gross disease did not result in increased levels of ADA in lymphocytes (Table II). There were no

TABLE I.—*Adenosine Deaminase Activities in Normal and Pathological States**

| Group (N) | Age range | Adenosine deaminase (u/$10^8$ cells) Lymphocytes | Granulocytes | Erythrocytes | Blasts |
|---|---|---|---|---|---|
| Normal (22) | 23–60 | 144±5 | 84±4 | 3·8±0·3 | — |
| Leukaemia | | | | | |
| Chronic lymphocytic, untreated (5) | 47–63 | 54±9 | 74±5 | 3·9±0·7 | — |
| Chronic lymphocytic, treating† (4) | 41–80 | 55±15 | 76±7 | 4·1±0·7 | — |
| Leukaemic reticuloendotheliosis* (2) | 40–51 | 82±28 | 88 | 3·7±0·2 | — |
| Acute lymphosarcoma-cell (1) | 30 | — | — | 5·9 | 2700 |
| Acute lymphocytic (7) | 3–17 | — | — | — | 860±312 |
| Acute myelogenous (6) | 19–83 | — | — | 5·1±0·6 | 513±89 |
| Lymphoma | | | | | |
| Hodgkin's, untreated (3) | 17–52 | 146±9 | 88±2 | 4·4±0·9 | — |
| Hodgkin's, treating† (3) | 38–72 | 246±46 | 89±11 | 4·5±0·2 | — |
| Hodgkin's, remission‡ (1) | 52 | 129 | 90 | 5·3 | — |
| Non-Hodgkin's, untreated (3) | 51–78 | 166±64 | 89±12 | 3·0±0·2 | — |
| Non-Hodgkin's, treating† (4) | 17–70 | 290±59 | 98±4 | 4·1±0·3 | — |
| Non-Hodgkin's, remission‡ (3) | 45–48 | 156±19 | 92±5 | 5·0±0·8 | — |
| Multiple myeloma, untreated (3) | 54–60 | 112±14 | 77±16 | 5·0±1·4 | — |

* The number of individuals in each group is indicated in parenthesis. Age range is given in years. Cells were obtained from peripheral blood and adenosine deaminase values are expressed as the mean ± s.e., except for leukaemic reticuloendotheliosis which is mean ± range. Patients were not receiving chemotherapy or radiation therapy except where indicated.

† Patients were receiving one or more cancer chemotherapeutic agents at the time blood was obtained. Specific information about the drugs used in one group of patients, CLL, presented in Table II. Because of the complexity of the chemotherapy and the lack of correlation between ADA and specific agents, further details about treatment are omitted.

‡ Patients in remission had received extensive chemotherapy or radiation therapy, but this had been discontinued at least one year before study. At the time blood was obtained there was no clinical evidence of recurrence of lymphoma.

TABLE II.—*Adenosine Deaminase Activities in Lymphocytes from Patients with Chronic Lymphocytic Leukaemia*

| Patient | Age (years) and sex | Duration of disease (years)* | Chemotherapy† | Adenosine deaminase‡ (u/$10^8$ lymphocytes) |
|---|---|---|---|---|
| 1 | 63, F | 2 | None | 46 |
| 2 | 47, M | 0 | None | 31 |
| 3 | 57, M | 1 | None | 42 |
| 4 | 50, M | 0 | None | 80 |
| 5 | 60, M | 0 | None | 71 |
| 6 | 61, M | 8 | Chl, Vin, Pred, 6TG | 37 |
| 7 | 62, M | 5 | Cyc, Vin, Pred, Chl, Pro | 92 |
| 8 | 80, M | 3 | Chl, Pred | 26 |
| 9 | 41, M | 5 | Cyc, Vin, Pred | 63 |

* Duration of disease is time from diagnosis by haematological evaluation and is not estimated from the patient's history of possible earlier signs and symptoms.

† Chemotherapeutic agents received by the patient as treatment of his leukaemia. Chl, chlorambucil; Vin, vincristine; Pred, prednisone; 6TG, 6-thioguanine; Cyc, cyclophosphamide; Pro, procarbazine.

‡ Assay of ADA in lymphocytes is very reproducible and repetitive determinations on blood specimens from the same donor are usually ± 10% of the mean (Coleman and Hutton, 1975*a*).

apparent correlations between therapy or duration of disease and ADA values, contrary to the report of Tung *et al.* (1974) who found that ADA levels increased toward normal in treated cases of CLL. ADA activities remained normal in granulocytes and erythrocytes of patients with treated and untreated CLL (Table I). Two patients with untreated leukaemic reticuloendotheliosis were studied. In one case the ADA activity in lymphocytes was very low (54 u/$10^8$ cells) whereas in the second patient the value was higher (110 u/$10^8$ cells) and closer to normal (Table I).

Blasts from patients with acute lymphocytic (ALL) and acute myelogenous (AML) leukaemia had levels of ADA activity 2 to 10-fold higher than lymphocytes of normal individuals (Table I). The activities observed in lymphoblasts varied more from patient to patient than did activities in myeloblasts, but in neither type of acute leukaemia did blasts have ADA activities comparable with those present in cells of normal peripheral blood. These observations disagree with the report of Zimmer *et al.* (1975) who found decreased levels of ADA in peripheral lymphoid cells from patients with acute lymphocytic leukaemia in relapse. The lymphoid cells consisted of a mixed population of lymphoblasts and lymphocytes. Our observations, however, agree with those of Smyth and Harrap (1975).

Lymphoma is frequently associated with impaired immunological function, especially defective cellular immunity. Whether lymphocytes have abnormal levels of ADA in lymphoma has not been reported. Lymphocytes from 3 untreated patients with Hodgkin's disease had normal ADA activities (Table I). Each of these patients had stage IIA disease. ADA activities in lymphocytes from people with untreated non-Hodgkin's lymphomata were variable, with values of 88 and 293 in lymphocytic lymphomata, 175 in a localized histiocytic lymphoma of the stomach, and 117 in a mixed histiocytic–lymphocytic lymphoma. Combination chemotherapy of both Hodgkin's and non-Hodgkin's lymphomata was associated with elevation of ADA activities in lymphocytes. Mean values $\pm$ s.e. for patients receiving chemotherapy were $246 \pm 46$ in Hodgkin's disease and $290 \pm 59$ in non-Hodgkin's lymphoma. Each of these values differs significantly ($P < 0{\cdot}01$, Student's "$t$" test) from normal. A number of different drug regimens were being employed in patients at various stages of their disease, including alkylating agents, vincristine and prednisone combinations, bleomycin, adriamycin and methotrexate. The observation that ADA values are elevated in lymphocytes of patients receiving chemotherapy for lymphoma appears consistent for commonly used drug regimens and contrasts with the values of ADA observed in patients with chronic lymphocytic leukaemia on chemotherapy. Four patients with lymphoma in clinical remission to whom chemotherapy had not been administered for at least one year had normal values of ADA activity. Lymphocytes had low levels of ADA in 3 people with untreated multiple myeloma.

Regardless of changes in ADA activity in lymphocytes, no consistent abnormalities were observed in erythrocytes or granulocytes (Table I). ADA values in extracts of granulocytes were remarkably constant in all the groups studied. There was greater variability in the red cell ADA values but in no group was there an extreme deviation from the control values. Because ADA activity is so low in erythrocytes compared with nucleated cells, minor degrees of contamination of erythrocytes with nucleated cells would elevate the apparent red cell ADA activity. Such contamination was monitored both by morphological examination of cell preparations and by differential particle counts before and after detergent treatment of cell suspensions. Contamination of erythrocytes with nucleated cells was generally less than 0·2% and ADA values in erythrocytes were corrected to eliminate the contribution of granulocytes and lymphocytes.

Since ADA activities were much higher in lymphoblasts of children with ALL than in normal lymphocytes, the effect of chemotherapy on ADA values was monitored in 5 children. Initial peripheral leucocyte counts ranged from 40,000 to 400,000/$\mu$l with 80–95% lymphoblasts. The specific activities of ADA in the blasts ranged from 349 to 2650 u/$10^8$ cells. The capacity of blasts from these patients to form E rosettes with sheep red blood cells has been reported (Coleman *et al.*, 1976). ADA values in blasts forming E rosettes were 549, 2650 and 345 u/$10^8$ cells; values in blasts not forming E rosettes were 562 and 349 u/$10^8$ cells. As seen in Fig. 1, there is a rapid decrease in ADA levels during induction of remission with vincristine and prednisone. By 21 days after induction therapy was begun, lymphoblasts had disappeared from the peripheral blood of each patient and the peripheral lymphocytes appeared morphologically mature. ADA values in these lymphocytes had returned to normal levels.

FIG. 1.—ADA activities in the lymphocyte–lymphoblast fraction from the peripheral blood of patients with acute lymphocytic leukaemia. Induction chemotherapy with vincristine and prednisone was begun on Day 1. Initially the lymphocyte fraction contained at least 80% lymphoblasts but this value decreased to 0% by Day 21, after which only mature lymphocytes were found. Different symbols represent different patients.

It seemed most likely that differences in the activity of ADA per lymphocyte in CLL or per lymphoblast in ALL compared with the activity in normal lymphocytes were due to changes in the level of the enzyme rather than to the presence of inhibitors or activators in homogenates. In order to investigate this possibility, a series of mixing experiments was performed using extracts of normal lymphocytes, lymphocytes from CLL and lymphoblasts from ALL. In all cases the enzymatic activities of the mixtures were as expected from addition of the separate activities with no evidence of inhibitors or activators.

Deviation from normal levels of ADA activity was consistently observed in lymphocytes of chronic lymphocytic leukaemia and in blast cells of acute myelogenous and acute lymphocytic leukaemias. This observation prompted an investigation of possible alterations in the molecular weight of the enzyme in association with these diseases since there are two species of ADA which differ in molecular weight (Akedo *et al.*, 1972; Osborne and Spencer, 1973). Cell extracts were sedimented through 5–20% sucrose gradients, as seen in Fig. 2. Recovery of ADA activity from the gradients was greater than 70%. The major species of ADA peaked in tube 7 with an estimated molecular weight of approximately 40,000. Lymphocytes from normal people (a) and from patients with CLL (b) generally contained a minor component of ADA with a high molecular weight. Activity in this component generally peaked in tube 19, with an estimated molecular weight of 200,000. This species comprises around 5% of the total ADA activity. Blasts from patients with AML (c) and ALL (d) had elevated activities of ADA. When sedimented through sucrose density gradients, ADA from these blasts was relatively homo-

FIG. 2.—Sucrose density gradient sedimentation of adenosine deaminase from sonicates of (a) normal lymphocytes, (b) lymphocytes of chronic lymphocytic leukaemia, (c) myeloblasts of acute myelogenous leukaemia, (d) lymphoblasts of acute lymphocytic leukaemia. Note the differences in scale of the ordinate for the different types of cells.

geneous with maximal activity in tube 7 and little or no activity in tube 19 (Fig. 2).

### DISCUSSION

The recent development of a convenient, sensitive radiometric method of assaying ADA in small numbers of cells from peripheral blood (Coleman and Hutton, 1975*a*) has facilitated our investigation of changes in ADA in haematological malignancies. One important conclusion from our work is that ADA values in lymphocytes vary in malignant diseases without associated changes in erythrocytes or granulocytes. Assays of one type of cell in the peripheral blood cannot be used to predict ADA levels in other types of cells.

ADA activity was low (approximately 35% of normal) in lymphocytes of patients with chronic lymphocytic leukaemia (Table II) but was normal in their erythrocytes and granulocytes. Scholar and Calabresi (1973) and Tung *et al.* (1974) have reported similar results in lymphocytes. The latter investigators found that ADA activity increased following chemotherapy, which is not supported by the data presented in Table II. In our experience, ADA activities in lymphocytes of newly diagnosed patients with chronic lymphocytic leukaemia and minimal disease are similar to those observed in patients receiving chemotherapy for far advanced disease. Chronic lymphocytic leukaemia is generally a B cell disorder and it is of interest that two other probable disorders of B cells, multiple myeloma and hairy-cell leukaemia (Catovsky *et al.*, 1974) are also associated with variable, but low, activities of ADA in peripheral lymphocytes (Table I).

Elevated values of ADA are regularly found in the lymphocytes of patients with lymphoma who are receiving intensive combination chemotherapy. This was unexpected since lymphocytes from patients with chronic lymphocytic leukaemia who were receiving similar drugs had low levels of ADA. Newly diagnosed patients with lymphoma, or those who have completed chemotherapy, rarely have abnormal values of ADA. One patient with a poorly differentiated lymphocytic lymphoma developed lymphosarcoma-cell leukaemia. Blasts from this patient's blood contained very high levels of ADA, resembling human thymocytes and cells of lymphoblastoid cell lines (Coleman and Hutton, 1975*b*).

ADA activity was elevated in blasts from patients with acute lymphocytic and acute myelogenous leukaemia. Differences in the specific activity per cell were insufficient and too variable to permit use of ADA as a biochemical marker for distinguishing the two types of blasts (Table I). We monitored ADA activity in the blood of 5 children during chemotherapy for acute lymphocytic leukaemia in order to see whether it could serve as a biochemical marker for the presence of abnormal cells in the peripheral blood. ADA values in peripheral lymphoid cells returned to normal as remission was achieved. There was no suggestion that ADA would be a useful biochemical marker of disease activity and completeness of remission.

The cause of alterations of ADA activity in lymphoproliferative diseases is not clear. Certainly variation in the stage of immunological maturation of the circulating lymphocyte may be important because leukaemic lymphoblasts, human thymocytes and lymphoblastoid cell lines all have high levels of ADA, when compared with normal mature circulating lymphocytes (Coleman and Hutton, 1975*b*). Elevated levels of ADA in lymphocytes would indicate changes in the degree of immunological activity compared to normal. Low values seem generally to be associated with abnormal B cell proliferation.

Our conclusion after assaying ADA in lymphocytes, granulocytes and erythrocytes of 45 patients with haematological disease is that the procedure is too laborious for the limited amount of information derived. Assay of the enzyme is rapid and inexpensive, lending itself to population surveys. It is the cell separation procedure that is time-consuming and expensive, prohibiting routine survey of populations. Cell separation techniques are necessary, however, because of the lack of correlation among erythrocyte, granulocyte and lymphocyte ADA levels except in case of profound hereditary deficiency due presumably to a structural mutation in the common catalytic unit. Even the lowest values of ADA observed in adult lymphocytes did not seem far enough below normal to cause disordered purine catabolism which could affect lymphocyte function and explain acquired immunodeficiency. This should be tested, however, by measuring the purine content of lymphocytes in chronic lymphocytic leukaemia.

This work was supported by Research Grant AM16013 from the National Institute of Arthritis, Metabolism and Digestive Diseases and by the United States Veterans Administration Project No. MRIS 3843–04.

### REFERENCES

Akedo, H., Nishihara, H., Shinkai, K., Komatsu, K. & Ishikawa, S. (1972) Multiple Forms of Human Adenosine Deaminase. Purification and Characterization of Two Molecular Species. *Biochim. biophys. Acta*, **276**, 257.

Boyum, A. (1968) Isolation of Mononuclear Cells and Granulocytes from Human Blood. *Scand. J. clin. Lab. Invest.*, **21**, 77.

Catovsky, D., Pettit, J. E., Galetto, J., Okos, A. & Galton, D. A. G. (1974) The B-lymphocyte Nature of the Hairy Cell of Leukemic Reticuloendotheliosis. *Br. J. Haemat.*, **26**, 29.

Coleman, M. S. & Hutton, J. J. (1975*a*) Micromethod for Quantitation of Adenosine Deaminase Activity in Cells from Human Peripheral Blood. *Biochem. Med.*, **13**, 46.

Coleman, M. S. & Hutton, J. J. (1975*b*) Terminal Deoxynucleotidyl Transferase and Adenosine Deaminase in Human Lymphoblastoid Cell Lines. *Expl cell Res.*, **94**, 440.

Coleman, M. S., Greenwood, M. F., Hutton, J. J., Bollum, F. J., Lampkin, B. & Holland, P. (1976) Serial Observations on Terminal Deoxynucleotidyl Transferase Activity and Lymphoblast Surface Markers in Acute Lymphoblastic Leukemia. *Cancer Res.*, **36**, 263.

GIBLETT, E. R., ANDERSON, J. E., COHEN, F., POLLARA, B. & MEUWISSEN, H. J. (1972) Adenosine Deaminase Deficiency in Two Patients with Severely Impaired Cellular Immunity. *Lancet*, ii, 1067.

OSBORNE, W. R. A. & SPENCER, N. (1973) Partial Purification and Properties of the Common Inherited Forms of Adenosine Deaminase from Human Erythrocytes. *Biochem. J.*, **133**, 117.

PARKMAN, R., GELFAND, E. W., ROSEN, F. S., SANDERSON, A. & HIRSCHHORN, R. (1975) Severe Combined Immunodeficiency and Adenosine Deaminase Deficiency. *New Engl. J. Med.*, **292**, 714.

SCHOLAR, E. M. & CALABRESI, P. (1973) Identification of the Enzymatic Pathways of Nucleotide Metabolism in Human Lymphocytes and Leukemic Cells. *Cancer Res.*, **33**, 94.

SMYTH, J. F. & HARRAP, K. R. (1975) Adenosine Deaminase in Leukaemia. *Br. J. Cancer*, **31**, 544.

TUNG, R., CONKLYN, M., SILBER, R. & HIRSCHHORN, R. (1974) Adenosine Deaminase in Lymphocytes from Normal Subjects and Patients with Chronic Lymphocytic Leukemia. *Am. Soc. Hemat.*, Abst 56, Atlanta, Ga, December.

ZIMMER, J., KHALIFA, A. S. & LIGHTBODY, J. J. (1975) Decreased Lymphocyte Adenosine Deaminase Activity in Acute Lymphocytic Leukemic Children and Their Parents. *Cancer Res.*, **35**, 68.